# Predictors of depression stigma

**DOI:** 10.1186/1471-244X-8-25

**Published:** 2008-04-18

**Authors:** Kathleen M Griffiths, Helen Christensen, Anthony F Jorm

**Affiliations:** 1 Centre for Mental Health Research, The Australian National University, Canberra, ACT, 0200, Australia; 2 ORYGEN Research Centre, Department of Psychiatry, University of Melbourne, Locked Bag 10, Parkville, Victoria, 3052, Australia

## Abstract

**Background:**

To investigate and compare the predictors of personal and perceived stigma associated with depression.

**Method:**

Three samples were surveyed to investigate the predictors: a national sample of 1,001 Australian adults; a local community sample of 5,572 residents of the Australian Capital Territory and Queanbeyan aged 18 to 50 years; and a psychologically distressed subset (n = 487) of the latter sample. Personal and Perceived Stigma were measured using the two subscales of the Depression Stigma Scale. Potential predictors included demographic variables (age, gender, education, country of birth, remoteness of residence), psychological distress, awareness of Australia's national depression initiative *beyondblue*, depression literacy and level of exposure to depression. Not all predictors were used for all samples.

**Results:**

Personal stigma was consistently higher among men, those with less education and those born overseas. It was also associated with greater current psychological distress, lower prior contact with depression, not having heard of a national awareness raising initiative, and lower depression literacy. These findings differed from those for perceived stigma except for psychological distress which was associated with both higher personal and higher perceived stigma. Remoteness of residence was not associated with either type of stigma.

**Conclusion:**

The findings highlight the importance of treating the concepts of personal and perceived stigma separately in designing measures of stigma, in interpreting the pattern of findings in studies of the predictors of stigma, and in designing, interpreting the impact of and disseminating interventions for stigma.

## Background

Recent evidence suggests that the stigma is a leading cause of concern for people with depression [1]. There have been many studies of stigma associated with mental disorders. However, only a minority of these have focused on what factors predict stigma associated with depression either among the general public or in people with depression. Such information may be critical to the successful design, tailoring and targeting both of public destigmatisation programs and interventions to reduce stigma in people with depression.

Studies that have investigated the predictors of depression stigma have produced apparently conflicting results. For example, some researchers have reported greater stigma among women than men [2,3] whereas others have reported the reverse [4,5] or no difference [6-9]. Similarly, studies have variously reported no effect of age [9-11], greater stigma among older people [2,6,12] and greater stigma among younger people [3,5,13].

Some of these apparent disparities in findings may result from the use of different populations; for example clinical samples comprising people with depression compared to representative population surveys or samples from different countries. Other apparent inconsistencies may stem from differences in the type of stigma under study or the use of measures that combine different types of stigma item. For example, some research has focused on participants' beliefs about the negative attitudes of others (*perceived stigma*) whereas other studies have examined what the participant believes personally about depression, described by Corrigan and his collaborators as *public stigma *[14] and by Griffiths and her collaborators as *personal stigma *[15]. One study focused on predictors of the depressed participant's view of their own depression (*self-stigma*) [11]. Yet other studies combine items tapping different stigma types into one scale [7,9,16]. It is conceivable that predictors of stigma differ not only according to the depression status of the respondent, but also as a function of the type of stigma under study and the scale used to measure it.

To date, studies reporting the predictors of personal/public stigma have been confined to nationally representative samples [2,5,6,10] and to our knowledge there are no published studies of predictors of this type of stigma among people with depression, although there has been one relevant study of self-stigma [11]. Conversely, studies of the predictors of perceived stigma have focused primarily on samples of people with depression [7]. Finally, although demographic predictors have been investigated in a number of studies, there is a paucity of information about other potentially important predictors. Many destigmatisation programs attempt to address stigma by improving public knowledge about mental disorders. However, little is known about the association between mental health literacy and stigma. Similarly, although there is some, inconsistent, evidence concerning the effect of levels of psychological distress on perceived stigma among clinical samples of people diagnosed with depression [7,12,16] there are no studies of the relationship between psychological distress and stigma in the community.

The aim of the present paper is to investigate a range of potential predictors for two types of depression stigma, Perceived and Personal, using parallel stigma scales containing identical items and using the datasets from three Australian samples. The first dataset was derived from a national household survey of attitudes, beliefs and knowledge about mental disorder including depression. It also contained data on respondent awareness of Australia's national depression initiative *beyondblue*, a government funded, independent, non-profit organisation whose aims include increasing community awareness and destigmatisation of depression. The second dataset was derived from a community survey of the attitudes and beliefs about depression of a randomly selected sample of residents in the Australian Capital Territory and the adjacent town of Queanbeyan in New South Wales. The third dataset comprised information provided by a psychologically distressed subset of the latter sample and included additional information about respondents' knowledge about depression (depression literacy).

## Methods

The methodology used in collecting data from the three samples surveyed in this paper is described briefly below. Further details can be found in previous publications [17,18] (Sample 1) and [15,19] (Samples 2 & 3). Ethics approval was granted for the collection of all datasets by the Human Research Ethics Committee of the Australian National University.

### Sample 1 (National)

Attitudes and information relevant to depression were collected from a total of 1001 Australian adults in a national face-to-face household survey during 2003 and 2004 of 3,998 Australian adults aged over 18 years. Households were sampled from 250 census districts covering all Australian States and both rural and metropolitan areas. Interviewers made up to five callbacks in metropolitan areas and three in rural areas. Response rate, computed as a percentage of the total number of contactable and physically available qualified respondents was 34%.

Respondents to the survey were presented with a vignette describing a person with depression (see Appendix 1). Half of the participants were administered a male version of the vignette and the other half a female version. The vignette satisfied DSM-IV and ICD-10 criteria for a Major Depressive disorder.

Respondents were asked a series of questions about the disorder depicted in the vignette. Stigma associated with the disorder was measured using a vignette version of the Personal and Perceived scales of the Depression Stigma Scale (DSS) [15,18]. The DSS-Personal subscale comprises 9 items and is concerned with the respondent's personal attitudes to depression and the DSS-Perceived stigma scale comprises 9 items assessing the respondents beliefs about the attitudes of others to depression. Scores for each subscale range from 0 to 36. Higher scores indicate greater stigma. The DSS scales have previously demonstrated acceptable internal consistency and test-retest reliability [15]. Respondents also completed a 5-item attitudinal Social Distance scale [20]. This scale measured self-reported willingness to make contact with the person in the vignette. Respondents rated their willingness to 1) move next door to the person in the vignette; 2) spend an evening socialising with the person; 3) make friends with the person; 4) work closely on a job with the person; and 5) have the person marry into the family. The respondent rated each item on a 4-point scale: 'definitely willing', 'probably willing', 'probably unwilling' and 'definitely unwilling'. A participants score on the scale was the mean rating across the 5 items (range 1 to 4) with higher scores indicating greater social distance.

Respondent capacity to recognise the problem depicted in the vignette as depression was evaluated [21]. Respondents were also asked if they had heard of Australia's national depression initiative, *beyondblue *[22], if they had 'ever had problems similar' to those of the character in the vignette, if their family had ever had such problems and if they had 'ever had a job that involved providing treatment or services' to a person with a problem like the character in the vignette. Information was collected about the respondent's sex, age category in years (18–19, 20–24, 25–29, 30–34, 35–39, 40–44, 45–49, 50–54, 55–59, 60–64, 65–69, 70–74, 75+), educational background, whether or not the respondent was born in Australia and the postcode of the respondent's residence. The latter was used to classify the locality of each participant according to the 2001 Australian Standard Geographical Classification (Major Cities, Inner Regional, Outer regional, Remote, and Very Remote) that applied to the majority of the population in the geographical region for the postcode. Respondents were also asked about their recall of media stories about depression and their current health status, as well as their views on which of a selection of options was the likely cause of the problem in the vignette, what might be helpful for treating the problem and their view on the likely prognosis for the person depicted in the vignette. However, these data are not the considered in the current paper.

Of the respondents, 59.9% were women, 22.5% had completed a Bachelor's degree or higher educational qualification, 67.8% resided in a major city and 26.2% were born outside of Australia. Median age was in the range 45 to 49 years. 33.1% reported that they had suffered from a problem similar to the character in the vignette (depression) and 15.1% indicated that they had suffered from 'depression' in the last month.

### Sample 2 (Local community)

This sample comprised 6,134 respondents who returned a screening questionnaire in a mailout to 27,000 individuals who were randomly selected from amongst registrants aged 50 years or less on the Canberra region electoral roll. Registration on the electoral roll is compulsory in Australia. Of the 6,134 respondents, 562 proved to be older than 50 years and were therefore excluded from the analyses reported here. Thus, the final sample size was 5,572. The Sample 2 survey did not involve a vignette. Stigma was measured using the Personal and Perceived Scales of the DSS [15]. Level of psychological distress was evaluated with the Kessler 10 (K10) [23] on a scale of 0 to 40, and exposure to depression with a modified version of the Level of Contact Report [24] on a scale of 1 to 12. Respondents were asked if they had 'ever been markedly depressed' and to indicate their age, gender, educational level, level of Internet access, whether they were receiving treatment from a mental health professional and their willingness to participate in an Internet intervention study.

The average age of the sample was 35.9 years (SD = 9.2), 65.2% were women, 44.3% had completed a tertiary qualification (Bachelor's degree or higher). 62.3% of the sample reported a history of depression.

### Sample 3 (Local community Distressed subset)

This sample comprised the subset of 487 Sample 1 respondents who were aged less than 50 years and who satisfied the eligibility criteria for participation in an Internet intervention trial. Criteria for inclusion were a K10 score of 12 or greater, access to the Internet, willingness to participate in the trial and not currently receiving treatment from a psychologist or a psychiatrist. In addition to responding to the survey questions administered to Sample 2, these respondents completed a second mail survey approximately 2 weeks after the first. The second survey comprised questions designed to evaluate the severity of participants' depressive symptoms (Center for Epidemiologic Studies Depression Scale (CES-D, [25]) and their level of dysfunctional thoughts using the Automatic Thoughts Questionnaire (ATQ) [26]. They also completed a 22-item Depression Literacy scale (D-Lit, maximum score = 22 [15]). In addition, the survey included some items that are not the subject of the current paper including the Cognitive Behaviour Therapy Literacy scale (CBT-Lit, [19]) and questions relating to Internet interventions and stage of change. The average age of this sample was 35.3 years (SD = 8.76), 43.5% had completed a tertiary qualification (Bachelor's degree or higher), and 72.1% were women. Average CES-D score was 21.6 (SD = 10.73) and 70.2% of the sample met the CES-D criteria (>16) for current depression. 93.2% of the sample had a self-reported history of depression.

#### Analyses

##### Psychometric characteristics of the stigma scales

In order to explore if the DSS subscales were valid, each of the datasets was subject to a principal component analysis (PCA) followed by varimax rotation with the aim of identifying a component structure for the DSS that was simple, reliable and interpretable. The estimated number of components in the scale was initially determined using both parallel analyses (95^th ^percentile) and the Velicer's Minimum Average Partial Method (MAP, [27]) using a script developed by O'Connor [28]. Kaiser-Meyer-Olkin measures of sampling adequacy and Barlett's tests of sphericity were conducted to ensure that the data were suitable for principal component analysis. Variables (items) were included if their component loadings were at least 0.32 (see Tabachnick and Fidell [29]). Consistent with recommendations by Velicer and Fava [27] and our aims, we opted to retain only well identified factors, rerunning the analyses and extracting fewer components where we identified less than three high loadings on a component (cutoff of 0.6).

##### Predictors of depression stigma

The above analyses suggested that the DSS Personal and DSS Perceived stigma scales were valid (see Results below). Therefore, the status of demographic and other variables as predictors of depression stigma were analysed separately for the DSS Personal stigma and DSS Perceived stigma scales for each sample (Samples 1 to 3) using a series of two-step hierarchical regression analyses with entry of demographic variables in the first step and other predictor variables in the second (see Tables 1 to 3). A hierarchical regression analysis was also conducted on the Social Distance scale data for Sample 1 using the same predictor variables as for the DSS scales. In the case of Sample 1, variables were entered as follows: age (12 category); sex (females = 0, males = 1); Education (Not tertiary = 0, Tertiary = 1); Country of birth (Australia = 0, Overseas = 1); Major city vs elsewhere (No = 0, Yes = 1); Awareness of *beyondblue: the national depression initiative *(No = 0; Yes = 1); History of depression (No = 0, Yes = 1); Family member with depression (No = 0, Yes = 1); Service provider (No = 0, Yes = 1); and Recognition of depression vignette (No = 0; Yes = 1). Survey 2 and 3 variables were entered as 'continuous' measures except for sex (females = 0, males = 1).

**Table 1:** Depression Stigma Scale (DSS) component loadings for Samples 1, 2 and 3.

	**Sample 1**		**Sample 2**		**Sample 3**	
**Item^#^**	**C1**	**C2**	**C1**	**C2**	**C1**	**C2**
14. Most people believe that it is best to avoid people with depression so that you don't become depressed yourself	**0.71**	0.05	**0.66**	0.10	**0.59**	0.07
11. Most people believe that depression is a sign of personal weakness	**0.71**	0.04	**0.72**	0.00	**0.74**	0.06
13. Most people believe that people with depression are dangerous	**0.70**	0.06	**0.66**	0.16	**0.63**	0.06
15. Most people believe that people with depression are unpredictable.	**0.66**	0.03	**0.62**	0.11	**0.55**	0.01
12. Most people believe that depression is not a real medical illness	**0.65**	-0.07	**0.69**	-0.09	**0.76**	-0.07
10. Most people believe that people with depression could snap out of it if they wanted	**0.61**	0.04	**0.66**	-0.02	**0.72**	-0.04
17. Most people would not employ someone they knew had been depressed	**0.57**	0.13	**0.64**	0.12	**0.65**	0.20
16. Most people would not tell anyone if they had depression	**0.56**	-0.13	**0.47**	-0.08	**0.49**	-0.07
18. Most people would not vote for a politician they knew had been depressed	**0.52**	0.22	**0.59**	0.14	**0.61**	0.18
						
2 Depression is a sign of personal weakness	-0.06	**0.75**	-0.08	**0.72**	-0.07	**0.63**
9 I would not vote for a politician if I knew they had been depressed	0.08	**0.70**	0.10	**0.69**	0.15	**0.71**
1. People with depression could snap out of it if they wanted	-0.14	**0.67**	-0.15	**0.68**	-0.17	0.**59**
3. Depression is not a real medical illness	-0.06	**0.75**	-0.13	**0.67**	-0.09	**0.65**
5. It is best to avoid people with depression so that you don't become depressed yourself	-0.03	**0.64**	0.06	**0.63**	0.03	**0.60**
8. I would not employ someone if I knew they had been depressed	0.06	**0.59**	0.08	**0.70**	0.09	**0.74**
4. People with depression are dangerous	0.20	**0.53**	0.12	**0.57**	0.04	**0.56**
7. If I had a problem like John's I would not tell anyone	0.15	**0.39**	0.14	**0.35**	0.16	**0.40**
6. People with depression are unpredictable	0.27	**0.34**	0.17	**0.45**	0.13	**0.37**

Loadings marked in bold exceed the 0.32 threshold

^#^Item wording for the survey used for Samples 2 & 3 are shown. The wording of the survey used for Sample 1 referred to 'a problem like John's/Mary's'. For example, "If I had a problem like John's I would not tell anyone".

**Table 2:** National adult sample (Sample 1): Hierarchical linear regression analyses (unstandardised regression coefficients) for variables predicting personal and perceived stigma and social distance

	**Depression Stigma Scale (DSS)**								**Social Distance Scale**			
		
	**Personal Stigma**				**Perceived stigma**							
			
	**Step 1**		**Step 2**		**Step 1**		**Step 2**		**Step 1**		**Step 2**	
						
	**B**	***p***	**B**	***p***	**B**	***p***	**B**	***p***	**B**	***p***	**B**	***p***
*Sociodemographic characteristics*												
Age^#^	0.35	0.0001***	0.23	0.006**	-0.15	0.003**	-0.13	0.01**	0.04	0.0001***	0.03	0.0001***
Male sex	1.64	0.0001***	0.85	0.006**	-0.61	0.08	-0.40	0.26	0.13	0.001**	0.08	0.043*
Education	-2.37	0.0001***	-1.50	0.0001***	0.02	0.96	0.33	0.44	-0.09	0.053	-0.04	0.42
Born overseas	0.90	0.017*	0.43	0.22	0.17	0.68	0.21	0.60	0.01	0.86	-0.03	0.56
Remoteness	-0.44	0.22	-0.23	0.48	-0.37	0.33	-0.40	0.29	-0.04	0.28	0.03	0.41
*Other*												
Aware of national depression initiative			-1.34	0.0001***			0.25	0.54			-0.07	0.10
History of depression			-0.96	0.005**			-1.12	0.004**			-0.11	0.008**
Service/treatment provider			-1.59	0.0001***			0.55	0.19			-0.07	0.12
Family member/friend with depression			-2.15	0.0001***			1.03	0.009**			-0.13	0.002**
Recognition of disorder			-1.46	0.0001***			0.40	0.29			-0.13	0.001**

R^2 ^(R^2 ^adjusted)	0.133 (0.128)		0.256 (0.249)		0.013 (0.008)		0.030 (0.020)		0.076 (0.071)		0.125 (0.116)	
R^2 ^change	0.133		0.123		0.013		0.017		0.076		0.049	
F change	29.33		31.55		2.59		3.30		15.74		10.65	
Sig F change	0.0001***		0.0001***		0.025		0.006**		0.0001***		0.0001***	

* p ≤ 0.05 **p ≤ 0.01 ***p ≤ 0.001. ^#^12 category

**Table 3:** Community sample aged 18 to 50 years (Sample 2): Hierarchical linear regression analyses (unstandardised regression coefficients) for variables predicting Personal and Perceived stigma

	**Personal Stigma (n = 5,335)**				**Perceived Stigma (n = 5,316)**			
		
	**Step 1**		**Step 2**		**Step 1**		**Step 2**	
				
	B	*p*	B	*p*	B	*p*	B	*p*
*Sociodemographic characteristics*								
Age	-0.01	0.22	0.00	0.97	0.02	0.033*	0.03	0.0001***
Male sex	2.16	0.0001***	1.91	0.0001***	-0.70	0.0001***	-0.55	0.0001***
Education	-0.30	0.0001***	-0.26	0.0001***	-0.16	0.0001***	-0.11	0.0001***
*Other*								
K10			0.08	0.0001***			0.13	0.0001***
Contact			-0.33	0.0001***			0.06	0.004**

R^2 ^(R^2 ^adjusted)	0.055 (0.054)		0.099 (0.098)		0.011 (0.011)		0.052 (0.051)	
R^2 ^change	0.055		0.044		0.011		0.041	
F change	102.62		130.56		20.25		113.726	
Sig F change	0.0001***		0.0001***		0.0001***		0.0001***	

* p < 0.05 **p < 0.01 ***p < 0.001

Potential interactions between predictor variables and stigma types were investigated using a series of Mixed Between-within subjects Analyses of Variance (Between variable = Predictor; Within Variable = Stigma Type). For the purposes of the ANOVAs, predictors were dichotomized to ensure comparability across surveys. Thus the 12-category age measure in the Sample 1 survey was collapsed into 4 categories (18 to 24 years; 25 to 49 years; 50 to 64 years; 65 years and above) and the continuous age measures employed in the survey for Samples 2 and 3 (age range 18 to 50 years) were dichotomized into (18 to 24 years; 25 to 50 years). Educational categories were categorised into Not tertiary = 0 or Tertiary = 1 for each survey. Psychological distress was categorized as either low to mild (K10 < 19) or moderate to severe (K10 = 20 to 40), Contact was dichotomised into high contact (score > 6) versus low contact (score < 7) and Depression Literacy was dichotomised into pass (>10) and fail (<11).

## Results

### Psychometric characteristics of the DSS stigma scales

#### Principal component analyses and internal reliability

The Sample 1 dataset satisfied the requirements for carrying out a PCA (Kaiser-Meyer-Olkin test = 0.81; Bartlett's test of sphericity p < 0.001). Both parallel analysis and MAO analysis suggested a three component solution for the Sample 1 dataset. However, the three-component PCA (varimax rotation) resulted in cross loadings on two of the items and one of the components did not include any loading of 0.6 or greater. A two component analysis yielded two interpretable factors (*Personal stigma *and *Perceived stigma*). In particular, all 9 items concerning the respondent's perceptions of others' attitudes loaded on component 1 and all 9 items concerning the respondent's own attitudes loaded on component 2 (see Table 1). Cronbach alphas were 0.78, 0.82, and 0.77 for the Total, Perceived and Personal components respectively indicating acceptable internal reliability.

The Sample 2 dataset satisfied the requirements for carrying out a PCA (Kaiser-Meyer-Olkin test = 0.80; Bartlett's test of sphericity p < 0.001). The parallel analysis suggested a four component solution for the Sample 2 dataset and the MAP analysis indicated a 3 component solution. However a principal component analysis (with varimax rotation) using the four component solution was unsatisfactory, as was the three component solution, each showing a substantial proportion of cross loadings. The two component solution was satisfactory (see Table 1). It comprised a Personal Stigma component (9 items) and a Perceived stigma component (9 items). The two component model accounted for 40.6% of the variance (21% Perceived,19.7% Personal). Cronbach alphas demonstrated acceptable internal consistency for both forms of the scale (DSS-Personal = 0.77; DSS-Perceived = 0.82).

The dataset for Sample 3 was a subset (n = 487) of that described by Griffiths et al. [30] (n = 525), but differed from the latter in that it did not include participants older than 50 years. The results of a two component solution with the reduced dataset are shown here for comparison. Kaiser-Meyer Olkin (0.78) and Bartlett tests (p < 0.001) indicated that the data satisfied the requirements for carrying out a PCA. The two component model accounted for 39.6% of the variance (Component 1/Perceived stigma = 21.4%; Component 2/Personal stigma = 21.4%). Cronbach alphas were 0.78, 0.82, and 75 for the Total, Perceived and Personal components respectively.

Based on the above analyses, it was concluded that it was appropriate to use the DSS Personal and DSS Perceived scales to examine separately the predictors of personal and perceived stigma.

#### Intercorrelations between scales

The correlation between the DSS-Personal attitude and Social Distance scores (Sample 1) was moderately high (r(998) = 0.53, p < 0.0001) providing evidence for the convergent validity of the DSS-Personal scale. Consistent with our previous report for Sample 3 data, the intercorrelations between the DSS-Personal Stigma and Perceived stigma were very small for both Sample 1 and 2 datasets (Survey 1: r = 0.12; Survey 2: r = 0.12). Similarly, the correlation between Social Distance and DSS-Perceived stigma was very small (Survey 1: r = 0.08).

### Predictors of stigma

#### Sample 1

Table 2 presents the results of the hierarchical regressions for the national survey for the DSS and Social Distance scales.

Personal stigma was significantly higher for those who were older, not-tertiary educated, and men. Personal stigma was also higher for people with lower levels of exposure to depression (those who had not previously experienced depression or who reported no depression among members of the family or who had not provided treatment or services to people with depression), in those who failed to recognise the person in the vignette as depressed, and in those who were unaware of Australia's national depression initiative, *beyondblue*. Remoteness of residence did not affect personal stigma levels. Being born overseas was associated with higher personal stigma independently of other demographic variables, but this effect disappeared when non-demographic predictors were included in the model.

The percentage of variance explained by the model for perceived stigma was very small (1.6% compared to 22.9% for personal stigma) and the pattern of findings for perceived stigma consistently differed from that for personal stigma. In contrast to the results for personal stigma, perceived stigma was lower (rather than higher) for older people and experience with a family member with depression predicted higher (rather than lower) perceived stigma. Moreover, in contrast to personal stigma, the demographic variables of sex, educational level and country of birth did not predict level of stigma and nor did knowledge of the disorder, awareness of Australia's national depression initiative (*beyondblue*) or being a service provider. The only similarities between the findings for the two types of stigma was that a self-disclosed history of depression predicted both lower perceived stigma and lower personal stigma and remoteness of residence predicted neither perceived stigma nor personal stigma.

This pattern of differences in predictors for the two types of stigma was broadly consistent with the results of mixed Within-Between ANOVAs as indicated by significant interactions between stigma type and each of the predictors (p < 0.05) except remoteness of residence (p = 0.60). Further analysis of the effect of age on stigma using Bonferroni comparisons revealed that participants aged 65 years or older demonstrated more personal stigma than the other age groups (p < 0.001 in each case), whereas the younger age groups did not differ significantly in personal stigma. There was a trend towards lower perceived stigma among participants aged 50 years and older compared to younger participants (uncorrected p-values: 20 to 24 years vs 50 to 64 years p = 0.035; vs 65 years and older p = 0.075; 25 to 49 years vs 50 to 64 years p = 0.011; vs 65 years and older p = 0.042) but these effects were not statistically significant after Bonferonni correction. There was no statistically significant difference between the two younger groups in perceived stigma either with or without Bonferroni correction (p > 0.05).

As might be expected, given the positive correlation between the two scales, the pattern of results for the regression analyses for social distance was broadly similar to those for DSS personal stigma. However, the effect of education was marginal (p = 0.053) and in contrast to the case for DSS-Personal stigma, country of birth, awareness of *beyondblue *and being a service provider did not independently predict lower social distance.

#### Sample 2

Table 3 summarises the results of the hierarchical regressions for the local community sample. The pattern of these results was broadly consistent with that for the national dataset, with personal stigma higher in men, those with less education and in those with less exposure to depression. Age in years failed to predict level of personal stigma, but there were no participants older than 50 years in Sample 2.

In contrast with personal stigma, females (rather than males) showed higher levels of perceived stigma and those with greatest self-reported contact with depression showed the greatest perceived stigma. Higher perceived stigma was associated with older age. As for Personal Stigma, lower educational levels and higher current psychological distress were associated with higher perceived stigma.

This pattern of differences in predictors for the two types of stigma was broadly confirmed by mixed Within-Between ANOVAs, as indicated by the significant interactions between stigma type and age, sex and the degree of self-reported contact (p ≤ 0.001). Consistent with the results of the hierarchical regression, participants in moderate to severe psychological distress showed both greater personal (t(1,5515) = 4.44, p < 0.001) and greater perceived stigma (t (1,5515)= 10.91, p < 0.001) than those with low to mild psychological distress. However, as evidenced by a significant interaction between current psychological distress and stigma type (p < 0.001), the discrepancy between the two groups was somewhat greater in the case of perceived stigma.

#### Sample 3

The third dataset enabled us to explore the relationship between depression literacy and stigma in those who, as a group, showed a high level of current depressive symptoms. Since all participants had elevated levels of psychological distress, of whom most (93%) had a self-reported history of depression, it was not appropriate to include contact as a variable in the analyses reported here.

Table 4 summarises the results of the hierarchical regressions for this high depressive symptom subgroup. As can be seen, controlling for demographic variables including education, higher personal stigma was associated with lower depression literacy and higher psychological distress in this group. Depressed men had higher personal stigma than depressed women. Personal stigma was also higher among those with less education, but this effect disappeared when psychological distress and depression literacy were added to the model. There was no association between age and personal stigma or perceived stigma. Nor was there an association between perceived stigma and depression literacy or gender. However, higher perceived stigma was associated with lower education and greater psychological distress.

**Table 4:** Psychologically distressed subset of community sample aged 18 to 50 years (Sample 3): Hierarchical linear regression analyses (unstandardised regression coefficients) for variables predicting Personal and Perceived stigma.

	**Personal Stigma (n = 472)**				**Perceived Stigma (n = 472)**			
		
	**Step 1**		**Step 2**		**Step 1**		**Step 2**	
				
	B	*p*	B	*p*	B	*p*	B	*p*
*Sociodemographic characteristics*								
Age	-0.004	0.88	-0.02	0.47	0.04	0.10	0.05	0.06
Male sex	3.33	0.0001***	2.99	0.0001***	0.59	0.24	0.60	0.23
Education	-0.25	0.014*	-0.13	0.20	-0.38	0.0001***	-0.32	0.002**
*Other*								
K10			0.11	0.011*			0.17	0.0001***
Depression literacy			-0.34	0.0001***			0.08	0.24

R^2 ^(R^2 ^adjusted)	0.093 (0.087)		0.152 (0.143)		0.039 (0.033)		0.077 (0.067)	
R^2 ^change	0.093		0.059		0.039		0.038	
F change	16.04		16.17		6.37		9.51	
Sig F change	0.0001***		0.0001***		0.0001***		0.0001***	

* p < 0.05 **p < 0.01 ***p < 0.001

Separate Mixed Within Between ANOVAs broadly confirmed the above pattern of findings. There was a significant interaction between stigma type and sex (p < 0.0001) and stigma type and depression literacy (p < 0.01) but no interaction between stigma type and psychological distress or education or age (p > 0.05). Participants with a greater level of psychological distress showed greater personal and perceived stigma, but in contrast to the Sample 2 results, this discrepancy was not significantly different for the two types of stigma.

## Discussion

To our knowledge, this is the first direct comparison between the patterns of predictors for perceived and personal stigma. It is also the first study to systematically investigate the predictors of personal stigma (including depression literacy) among people with a high level of depressive symptoms.

Notably, the pattern of findings for personal stigma was very similar for the national, community and depressed samples (see Table 5). For each of these samples, personal stigma was higher among men, and those with less education, greater psychological distress, and lower depression literacy. In addition, a lower level of self-reported prior contact with depression was associated with higher stigma in both the national and community samples. These findings differed from those for perceived stigma in a number of important ways. First, the percentage of variance in perceived stigma that was explained by predictors was very small, particularly for the national survey. Secondly, the pattern of findings for perceived stigma showed somewhat greater inconsistency across and within the three samples. Thirdly, some of the associations were reversed in comparison with the pattern noted for personal stigma. Thus, with some exceptions, self-reported contact tended to be associated with higher perceived stigma, whereas it was associated with consistently lower personal stigma. Similarly, in contrast to the pattern for personal stigma, depression literacy did not affect level of perceived stigma and perceived stigma was not higher among men, being either less (community sample) or equal to that for women. Findings for age also differed for the two types of stigma. The only consistently similar finding for personal and perceived stigma was that current psychological distress was a predictor for both stigma types.

**Table 5:** Summary of variables that predict depression stigma and attitudinal social distance for each of the three surveys.

**Sample**	**Older**	**Males**	**Lower Education**	**Rural**	**Born overseas**	**Not aware beyond-blue**	**Less contact**	**More distress (K10)**	**Lower depression literacy**
*Personal stigma*									
National (Sample 1)	higher (>= 65 yrs)	higher	higher	=	higher	higher	higher		higher^a^
Local community (Sample 2)	= (sample < 50 yrs)	higher	higher				higher	higher	
Depressed community (Sample 3)	= (sample < 50 yrs)	higher	higher (model 1)					higher	higher^b^
*Perceived stigma*									
National (Sample 1)	lower	=	=	=	=	=	lower (consumer) higher (carer)		=^a^
Local community (Sample 2)	higher	lower	higher				lower	higher	
Depressed community (Survey 3)	=	=	higher					higher	=
*Social distance*									
National (Sample 1)	higher	higher	higher (p = 0.053)	=	=	=	higher (except NS service provider)		higher

^a ^Recognition of depression item; ^b ^D-Lit; yrs = years; NS = Not significant

The finding that the pattern of predictors differed for personal/public and perceived stigma demonstrates the importance of treating these concepts separately, not only in designing measures of stigma (some researchers combine perceived and personal stigma items in one scale) and in interpreting the pattern of findings in studies of the predictors of stigma, but also in designing, interpreting the impact of and disseminating interventions for stigma.

On the other hand, the evidence that the pattern of findings for personal stigma in people with depression is similar to that for people in the broader community, demonstrates for the first time that similar risk factors for personal stigma apply whether the person is depressed or not. Thus the current findings suggest public destigmatisation programs targeted at those who are less educated, male, or born overseas or who have higher current psychological distress and lower depression literacy may also be effective in targeting those people with depression who are most at risk of holding stigmatising (personal) views. However, since personal stigma may be associated with a number of negative clinical outcomes including reduced adherence to appropriate treatments, such programs should not be restricted to public health campaigns but should also be applied at an individual level by health practitioners when providing treatment to people with depression.

**Figure 1:**
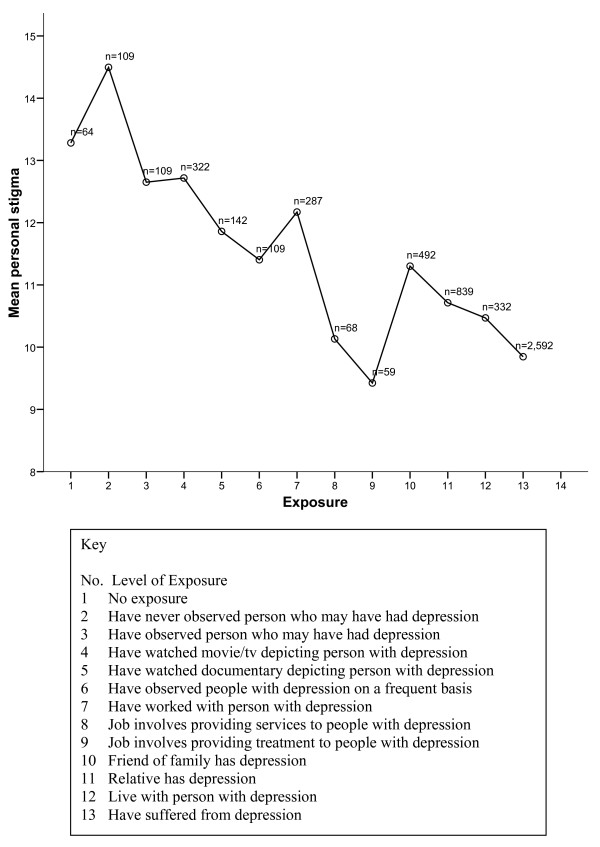
Relationship between Personal stigma and level of contact (based on modified Level of Contact Report [24]).

As has been reported in previous studies [2,5], self reported contact with depression was associated with lower personal stigma and lower social distance. However, family members (Sample 1) and members of the general community with higher levels of contact with depression (Sample 2) reported higher levels of perceived stigma. It is not clear whether this effect results from a greater exposure by those in closer contact to instances of stigma and discrimination directed at people with depression or a greater sensitivity to such events or both. By contrast, people self-reporting a history of depression in the national sample showed both lower personal stigma/social distance and lower perceived stigma. If exposure to stigma were critical in yielding higher levels of perceived stigma, it might have been expected that, like family members, this group would show higher perceived stigma. It may be that participants in the face-to-face survey who were willing to report a history of depression were those who perceived less stigma in the community and hence less reason to conceal their history of illness. Equally, it is possible that the association between *personal *stigma/social distance and self-reported depression was affected by the respondents' willingness to self-disclose the presence of depression, particularly given that one of the personal stigma items was "If I had a problem like John's I would not tell anyone". Finally, the finding that providers of mental health services show less personal stigma in both the national and community samples is of interest given the often cited claim that stigma is high among providers. In fact, health providers ranked lowest on personal stigma of all levels of contact for Sample 2 (see Figure 1).

We found that depression literacy was associated with lower personal stigma in the depressed group and that correctly recognising depression was associated with less personal stigma and lower social distance in the national sample as was knowledge of *beyondblue*, Australia's national depression initiative. These findings are contrary to those of Lauber et al.[2], who reported higher levels of social distance among participants who recognised a depression vignette as depicting a 'mental illness' in a national Swiss survey. They also differ from Angermeyer and Matschinger's findings from a representative survey in Germany, which found that labelling a depression vignette as either depression or another mental illness was unrelated to social distance [31]. It is unclear if the discrepancies between these findings and our own arise as a result of cultural differences or some other factor such as the use of different tasks of recognition and literacy. It is encouraging that in our Australian sample, those who recalled the national depression initiative, and those with better depression knowledge held less stigmatising attitudes. However, it is not possible to determine if this knowledge leads to lower personal stigma or if lower personal stigma leads to improved knowledge.

We have previously reported substantially higher levels of perceived compared to personal stigma in surveys 1 and 3 [15,18]. Griffiths et al. [18] suggested that this pattern might be attributable to an over-estimation of the prevalence of stigmatising beliefs in the community due to improved awareness of depression resulting from initiatives such as *beyondblue*. However, the current analysis yielded no evidence that awareness of *beyondblue *was associated with greater perceived stigma (p = 0.66). It is still possible that general media exposure about depression, some of which may have been triggered or promoted by public health initiatives such as *beyondblue *has led to an overall increase in perceived stigma. Indeed, we have previously reported that there has been an increase over a 7 year period in the belief that a person with depression would be discriminated against, particularly in those Australian States with greatest exposure to *beyondblue *[22].

We found that level of current psychological distress was associated both with higher personal stigma and higher perceived stigma in each of the samples. This is consistent with findings from two other studies of a relationship between level of depressive symptomatology and stigma where the latter was evaluated with a measure comprising mixed personal and perceived items in one case [9] and primarily perceived items in the other [16]. Two other studies found no effect of depression severity on measures comprising perceived [12] and primarily perceived stigma items respectively [7]. In the current study, when CES-D scores were substituted for K10 scores in the hierarchical regression, higher CES-D depressive symptoms were associated with higher perceived stigma (p = 0.001) but not higher personal stigma (p = 0.21). This raises the possibility that the K10 is tapping a factor other than level of depressive symptoms that is important in personal stigma. Substitution for the K10 by the ATQ, a measure of dysfunctional thoughts, produced a similar pattern to the CES-D suggesting that cognitive distortions may be particularly important in the perceptions of others' attitudes. Further investigation of these factors is required.

The finding that the level of personal and perceived stigma among rural residents is the same as that among their city counterparts challenges the common belief that level of stigma associated with depression is higher among rural residents than those in the city [32,33]. Significantly, this was true both controlling for and not controlling for demographic and other variables. Apart from the current study, there is little empirical evidence concerning the relative prevalence of stigma in rural and city residents. One previous US study did investigate perceived stigma among local convenience samples of city and rural residents with and without depressive symptoms in two adjacent counties [33]. In that study there were no rural-urban differences in perceived stigma associated with the condition of depression itself or with treatment for the overall sample. Rural residents with depressive symptoms did show a non-significant trend towards greater perceived treatment stigma, but this effect disappeared after controlling for education.

## Limitations

There are a number of limitations of the present study. First, as has been noted in our previous reports, response rate for the surveys is of some concern. Second, not all measures were used in each survey, so that it was not possible to comprehensively compare all predictors across surveys. Third, this study evaluated personal and perceived stigma in a group of people with high levels of psychological distress rather than people with diagnosed depression. However, almost all self-reported a history of depression and 70% exceeded the clinical cut-off score for depression on the CES-D. In addition, the findings or trends in the findings were broadly similar when only those people with a CES-D scores above 16 were included in the analysis. However, there is a need to repeat the study using suitable diagnostic evaluations. Fourth, the predictors did not explain a large percentage of the variance, particularly in the case of perceived stigma. In recognition of this, the practical suggestions in this paper relate primarily to personal stigma. Finally, this is a cross-sectional study and thus cannot be taken to provide definitive evidence of causal relationships between the predictors and stigma.

## Conclusion

The findings from this study suggest that in addition to delivering broad-based programs to reduce personal stigma, there may be value in targeting and tailoring programs to reduce personal stigma among men, older people, and those with lower educational levels and those born overseas. Consideration should also be given to developing destigmatisation programs for people with symptoms suggestive of psychological distress and to improving depression literacy. The effects of sociodemographic and other factors on perceived stigma were very small. However, perceived stigma is very high both in people with depression and the general public and may impact on help seeking. This suggests the appropriateness of programs at all levels (national and clinically targeted) designed to reduce perceived stigma, including those publicising the actual levels of personal stigma.

## Competing interests

The author(s) declare that they have no competing interests.

## Authors' contributions

KMG wrote the paper, conducted the statistical analyses and co-designed the Australian survey. HC Co-designed the Australian survey and edited the paper. AFJ Co-designed the Australian survey and edited the paper.

## Appendix: The depression vignette (John version)

John is 30 years old. He has been feeling unusually sad and miserable for the last few weeks. Even though he is tired all the time, he has trouble sleeping nearly every night. John doesn't feel like eating and has lost weight. He can't keep his mind on his work and puts off making decisions. Even day-to-day tasks seem too much for him. This has come to the attention of his boss, who is concerned about John's lowered productivity.

## Pre-publication history

The pre-publication history for this paper can be accessed here:


